# miR-612 suppresses stem cell-like property of hepatocellular carcinoma cells by modulating Sp1/Nanog signaling

**DOI:** 10.1038/cddis.2016.282

**Published:** 2016-09-29

**Authors:** Yang Liu, Dong-Li Liu, Li-Li Dong, Duo Wen, Dong-Min Shi, Jian Zhou, Jia Fan, Wei-Zhong Wu

**Affiliations:** 1 Liver Cancer Institute, Zhongshan Hospital, Fudan University, Key Laboratory of Carcinogenesis and Cancer Invasion, Ministry of Education, Shanghai 200032, China; 2 Department of Head and Neck Surgery, Fudan University Shanghai Cancer Center, Shanghai 200032, China; 3 Institute of Biomedical Sciences, Fudan University, Shanghai 200032, China

## Abstract

In our previous study we found that miR-612 negatively regulated stem cell-like property and tumor metastasis of hepatocellular carcinoma cells (HCC). In this study, we try to elucidate underlying mechanism of the regulation, and find that miR-612 inversely modulate the mRNA and protein level of epithelial cell adhesion molecule as well as CD133, negatively regulate the numbers and sizes of tumor spheres, directly inhibit the protein level of Sp1, and subsequently reduce transcription activity of Nanog. Of importance, the higher levels of Sp1 and Nanog in biopsies are the more unfavorable prognoses of HCC patients are found after tumor resection. Taken together, miR-612 has a suppressive role on HCC stemness via Sp1/Nanog signaling pathway.

Hepatocellular carcinoma (HCC) is the fifth most common malignance in the world.^1^ And its death rate has gradually risen in both sexes for the past 10 years.^2^ Tumor recurrence and metastasis are the two landmark events, which make tumor a locally growing disease into a systemic and life-threatening one. Owing to its high incidence of metastasis, HCC has been the third leading causes of cancer death in China.^3^ Therefore, it is imperative to find out metastatic biomarkers and cells for HCC early diagnosis and intervention.

Recently cancer stem cells (CSCs) are widely accepted as the initiating cells or seeds of tumor, which have been confirmed not only related to carcinogenesis but also to tumor progress. Like normal stem cells, CSCs usually possess self-renewal and multi-lineage potentials, and finally resulting in tumor heterogeneity, metastasis and drug resistance.^4^ Although intensive effects have been made in the past decades, there still no unique phenotype of HCC CSCs was identified. However, HCC cells expressing high level of a specific antigen, such as PROM1 (CD133),^5^ THY1 (CD90),^6^ epithelial cell adhesion molecule (EpCAM)^7^ and CD24,^8^ often exhibit cancer stem-like property, attribute to tumor metastasis and result in a dismal clinical prognosis. So, to define the roles of these side populations in HCC growth and metastasis was deadly needed at present.

MicroRNAs (miRNAs) are endogenous small noncoding RNAs, which can regulate gene expression at post-transcriptional level.^9, 10, 11^ Like protein-coding mRNAs, miRNAs have been proven as key players in different dimensions in CSCs reprogramming and induction.^12, 13^ Several miRNAs were revealed having distinctively roles on self-renewal and pluripotent maintaining of CSCs.^14^ In our previous studies, miR-612, a pleiotropic noncoding RNA, was found to suppress HCC stemness by reducing tumorsphere number and size as well as clone formation in soft agar, relieving drug resistance to cisplatin and 5-fluorouracil, and inhibiting HCC local invasion and distant metastasis through a EMT-regulated signal pathway.^15, 16^ Here we reported that Sp1/Nanog signaling pathway was another novel one, directly modulated by miR-612 in HCC CSCs reprogramming.

## Results

### MiR-612 inhibited EpCAM and CD133 expression in HCC

**Figure 1:**
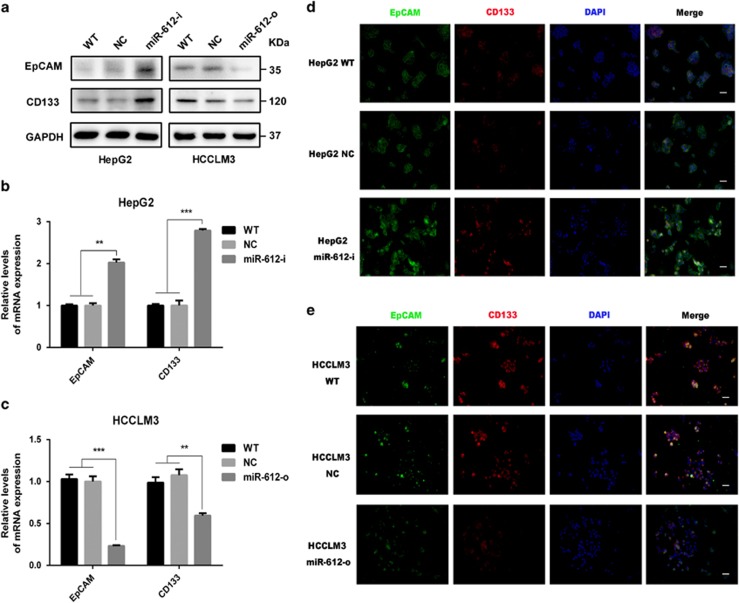
The inverse correlation between miR-612 and EpCAM, CD133. (**a**) Western blot analysis. (**b**) qRT-PCR. (**c**) Immunofluorescence staining to evaluate the expression levels of EpCAM, CD133 in transfected with miR-612 mimics, inhibitor and negative control in HCCLM3 and HepG2 cells. Scale bar: 50 *μ*m. Statistical analysis by Student's *t*-test (**P<0.01, ***P<0.001)

In the latest study we found that miR-612 significantly attenuated the forming abilities of non-attached tumor spheroids, decreased chemo-sensitivity against cisplatin and 5-fluorouracil, hampered HCC growth and metastasis in NOD/SCID mice.^16^ These findings imply that miR-612 could be a pivotal regulator of HCC stemness. To study its roles deeply, two postulated biomarkers of CSCs, EpCAM and CD133, were surveyed in miR-612 operated cells.^5, 7, 17^ After HepG2, a cell line with high endogenous miR-612 level and HCCLM3, a cell line with low endogenous miR-612 level were treated with miR-612 inhibitor (miR-612-i) and mimic (miR-612-o), respectively, the protein levels of EpCAM and CD133 in miR-612-i HepG2 cells were remarkably upregulated, whereas significantly downregulated in miR-612-o HCCLM3 cells compared with those wild type (WT) and scramble transfected cells (negative control, NC) (Figure 1a). Similar results were observed in the mRNA levels. In detail, treatment with miR-612-i resulted in significant increase of EpCAM by 1.0-fold (*P*<0.01, Figure 1b) and of CD133 by 1.7-fold (*P*<0.001, Figure 1b) in HepG2 cells. But the mRNA levels of EpCAM, CD133 were decreased by 0.7-fold (*P*<0.001) and 0.3-fold (*P*<0.01), respectively, in miR-612-o HCCLM3 cells (Figure 1c). In addition, the fluorescence intensities of both biomarkers were strikingly increased in miR-612-i cells (Figure 1d), and vice versa in miR-612-o cells (Figure 1e). Together, these data reminded us once again that miR-612 did have a negative role on CSCs programming in HCC.

### miR-612 negatively regulated Nanog in HCC

**Figure 2:**
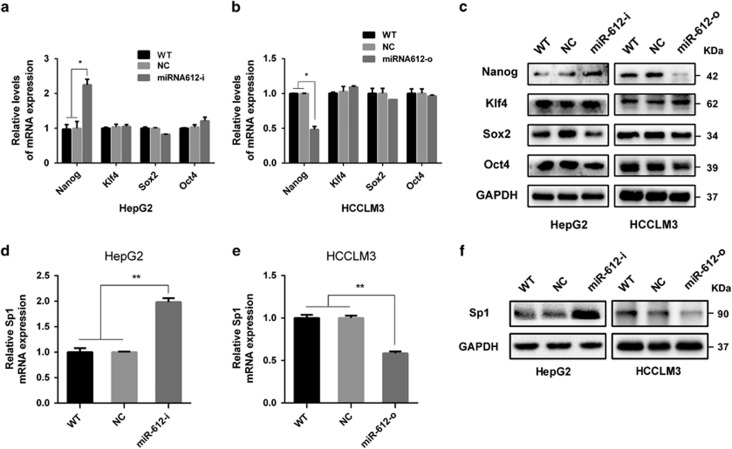
The mRNA and protein levels of transcription factor and Sp1 in HepG2, HCCLM3 with miR-6120-i, miR-612-o, respectively. (**a** and **b**) qRT-PCR. (**c**) Western blot analysis of Nanog, Klf4, Sox2 and Oct4 in miR-612-o HCCLM3 and miR-612-i HepG2 cells, respectively. (**d** and **e**) qRT-PCR. (**f**) Western blot analysis of Sp1 after indicated treatments, respectively. Statistical analysis by Student's *t*-test (*P<0.05, **P<0.01)

Like embryonic stem cells (ESC), several transcriptional factors regulating CSCs self-renewals and pluripotent properties were found in various cancers.^18, 19^ They are Oct4, Sox2, Klf4 and Nano and so on whose upregulation in cancer cells are usually related to stemness maintaining, tumor local invasion and distant metastasis.^20^ To bring insight into the roles of miR-612, mRNA and protein levels of above four transcriptional factors were first detected in miR-612-i HepG2 and miR-612-o HCCLM3 cells, respectively. As expected, deceasing miR-612 in miR-612-i HepG2 cells would markedly upregulate Nanog levels both in mRNA and protein more than twofold (*P*<0.01; Figures 2a and c), whereas increasing miR-612 in miR-612-o HCCLM3 cells would significantly downregulated Nanog levels by 50% (*P*<0.05; Figures 2b and c). Almost no significantly changes of Oct4, Sox2 and Klf4, were observed in miR-612 modulated cells. These results implied that miR-612 exerted its CSCs reprogramming possibly via Nanog.

### miR-612 suppressed Nanog by Sp1

Next, we tried to establish a direct role of miR-612 on Nanog expression using several bioinformatics methods. But no classical miR-612 seed sequences could be predicted successfully in Nanog mRNA using miRanda, TargetScan and miRWalk. However, such a seed sequence was found in Sp1 mRNA, a former confirmed upstream factor of Nanog. The finding hinted us that an indirect way of miR-612 on Nanog expression probably existed in HCC cells.

**Figure 3:**
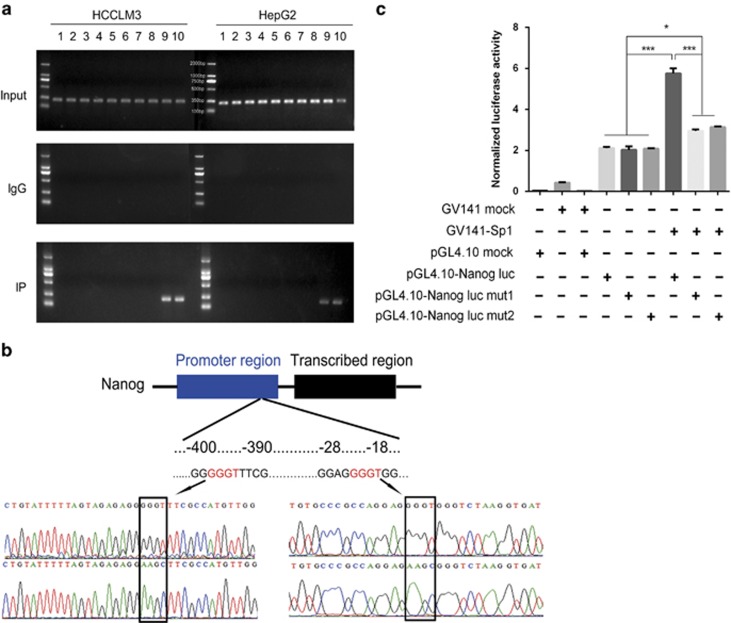
Sp1 binds to the promoter of Nanog. (**a**) Chromatin immunoprecipitation in both HepG2 and HCCLM3 cell lines. (**b**) Schematic diagram of the dual luciferase protein target reporter vector with wild type (upper panel) or mutant (lower panel) of Nanog and confirmed by DNA sequencing. (**c**) Luciferase activity was assayed in miR-612-o HCCLM3 cells. Data are mean±S.D. (*n*=3) and are representative of three independent experiments

As two putative Sp1-binding sites were found in 5'-flanking region of *nanog*,^21^ Chip-PCR assays were performed using 10 paired primers against further upsteam of *nanog* ORF per 300 nt. Actually, Sp1 was indeed recruited to the promoter of the gene in both HCCLM3 and HepG2 cell lines by the ninth and tenth primers, which indicated that the binding sites lied in 600 nts upstream region from ORF. And the binding intensities of endogenous Sp1 were about three times higher in HCCLM3 than these in HepG2 cells (Figure 3a). To further confirm, a dual luciferase reporter assay with WT (upper panel, Figure 3b) or mutant (lower panel, Figure 3b) of Nanog, verified by DNA sequencing, was constructed, respectively (Figure 3b). Twenty-four hours after co-transfected with Sp1 in HCCLM3 cells, the luciferase activities with WT promoter (−400~−390 or −28~−18 nts) were almost two- to threefold higher than these of mutants (5.75±0.25 *versus* 2.96±0.07 and 3.14±0.04; *P*<0.001) and three NCs (2.11±0.06, 2.02±0.17 and 2.08±0.08; *P*<0.001; Figure 3c). In addition, the luciferase activities with either mutant were 1.5-fold higher than NCs (*P*<0.05). All these results indicated that Sp1 could promote *nanog* expression directly by either binding site of the gene.

**Figure 4:**
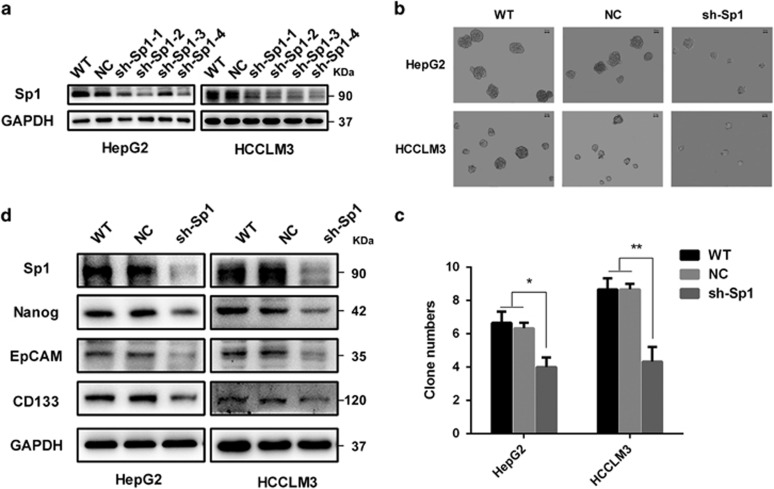
Sp1 suppresses the stemness of HCC *in vitro*. (**a**) Western blot analysis to evaluate the expression levels of Sp1 in HepG2, HCCLM3 wild-type (WT), negative control (NC) and Sp1 knockdown (shRNA1, shRNA 2, shRNA 3, shRNA4) cells. (**b** and **c**) Representative images and statistical results of HepG2 and HCCLM3 tumor spheres (>50 *μ*m) after indicated treatments (*n*=3; scale bar: 50 *μ*m). (**d**) Western blot analysis of Nanog, EpCAM, CD133 in HepG2, HCCLM3 wild-type (WT), negative control (NC) and Sp1 knockdown (shRNA4) cells. Statistical analysis by paired *t*-test (*P<0.05, **P<0.01)

To explore the effect of Sp1 on HCC stemness, four shRNA plasmids, sh-Sp1-1, sh-Sp1-2, sh-Sp1-3 and sh-Sp1-4 (Supplementary Table S1), were constructed, and their interfering efficiency measured by western blot assays. Among them, sh-Sp1-4 had the most interfering efficiency against Sp1 in both HCC cell lines (Figure 4a), thus chosen to use in the following lose-of-function study. Indeed, the average numbers of tumor spheres in HepG2 cells pretreated with sh-Sp1-4 were significantly decreased than those in control and WT cells, which were 4.0±0.8, 6.3±0.5, 6.8±0.7, correspondingly (*P*<0.05). Similar results were observed in HCCLM3 cells, which were 4.2±1.0, 8.8±0.2, 8.9±0.4 in shRNA, control and WT cells, respectively (*P*<0.01) (Figures 4b and c). Also, the sizes of tumor spheres in sh-Sp1-4-treated cells were remarkably smaller than those in the control and WT groups in both HCC cell lines (Figure 4b). More interestingly, EpCAM and CD133, two putative phenotypes of CSCs, were decreased significantly in both sh-Sp1-4-treated HCC cells (Figure 4d). Simultaneously, the level of Nanog was obviously suppressed in sh-Sp1-4-treated HCC cells. Taken together, Sp1 was a direct upstream factor on *nanog* transcription, whose downregulation would block stem cell reprogramming in HCC.

### Sp1 is a direct target of miR-612

**Figure 5:**
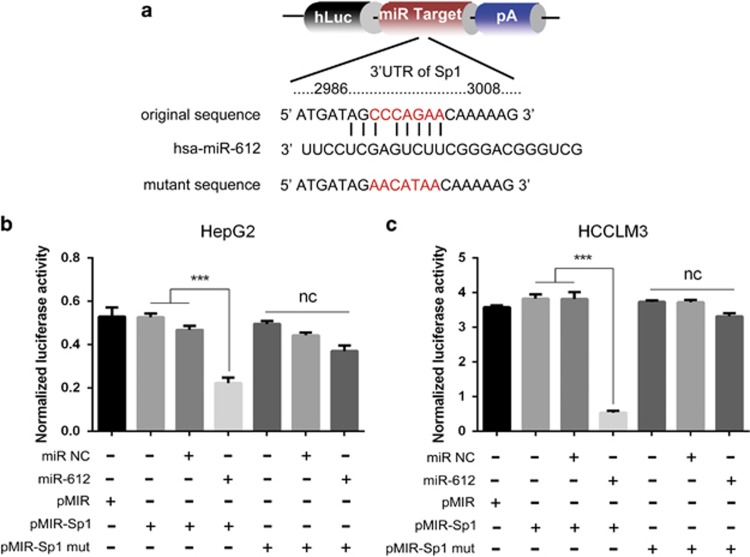
Direct regulation of miR-612 on Sp1 expression. (**a**) Schematic diagram of the dual luciferase miRNA target reporter vector. (**b** and **c**) Luciferase activity was assayed in miR-612-i HepG2 cells and miR-612-o HCCLM3 cells. Data are mean±S.D. (*n*=3) and are representative of three independent experiments. Statistical analysis by Student's *t*-test (***P<0.001)

**Figure 6:**
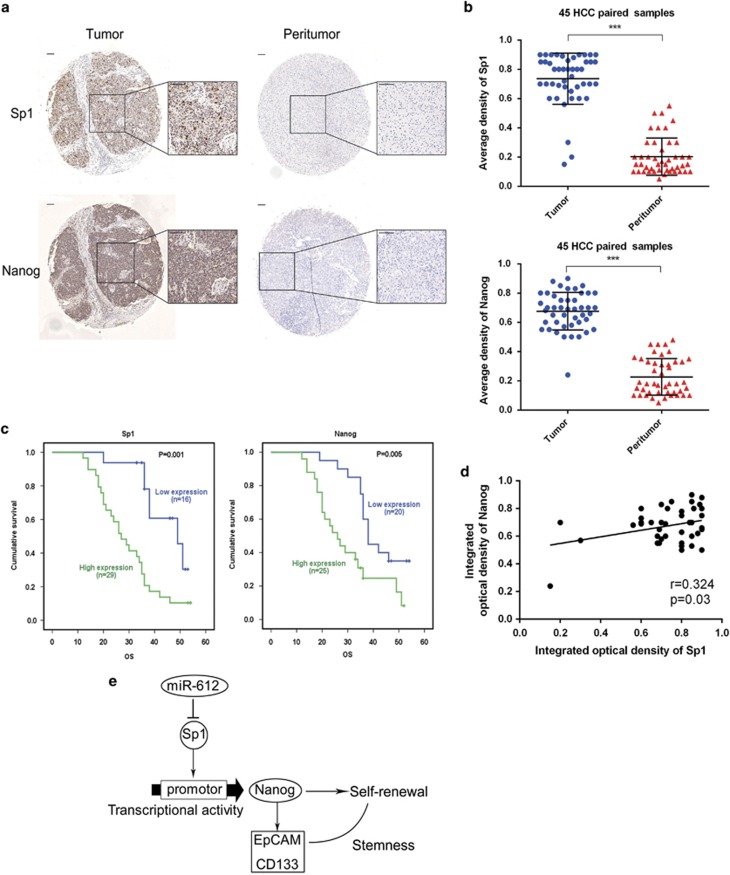
Clinical validation of Sp1 and Nanog in HCC patients. (**a** and **b**) Representative positive and negative Sp1 and Nanog expression in HCC tissues and their paired adjacent non-HCC tissues in immunohistochemistry. Bars: (left) magnification × 100, (right) magnification × 400. (**c**) Kaplan–Meier analysis of overall survival in HCC patients using SPSS 19.0. (**d**) Correlation analysis between Sp1 and Nanog. (**e**) Diagram of miR-612/Sp1/Nanog axis suppresses the stemness

Usually miRNAs regulate gene expression at post-transcriptional level by directly targeting seed sequence of the gene. As a predicted binding sequence existed in Sp1 mRNA, we wonder whether the suppressive effects of miR-612 on HCC CSCs were achieved by Sp1, a vital transcription factor functionally related to cell proliferation, differentiation, apoptosis, drug resistance and metastasis.^22, 23^ Definitely, Sp1 was significantly suppressed in miR-612-o HCCLM3 compared with WT and NC cells (1.98±0.07 *versus* 1.00±0.06 and 1.00±0.06; *P*<0.01), whereas remarkably increased in miR-612-i HepG2 compared with WT and NC cells (0.55±0.02 *versus* 1.00±0.03 and 1.01±0.02; *P*<0.01). The similar was in protein levels. (Figure 2d–f). Furthermore, dual luciferase reporter assays were performed to test its direct role of miR-612 on Sp1 in both HCC cells with WT sequence (2986–3008 nts) or mutant (Figure 5a). Twenty-four hours after co-transfected with miR-612 mimics or scramble (control), the luciferase activities were significantly inhibited by 50% in WT transfected HepG2 cells (*P*<0.001; Figure 5b) and 86.8% in HCCLM3 cells (*P*<0.001; Figure 5c), whereas no significantly change in mutant transfected cells. All the results indicated that miR-612 suppressed HCC stemness by directly targeting Sp1 (Figure 6e).

### Sp1 and Nanog levels in HCC patients

To investigate the profiles of Sp1 and Nanog in tumor tissues, 45 HCC patients were enrolled in the study and their surgical biopsies were collected with informed consent. The clinic pathological features of these patients are listed in Table 1. Sp1 in 42/45 tumor tissues and Nanog in 44/45 tumor tissues were evidently evaluated than these in corresponding para-tumor tissues when analyzed by IHC. Both Sp1 and Nanog were mainly located in the nucleus, although the latter was also observed faint in cytoplasm (Figure 6a). Meanwhile, a positive correlation between Sp1 and Nanog in tumor tissues was found (*r*=0.324, *P*=0.03; Figure 6d). The average density of Sp1 in cancerous tissues was 0.74±0.17, which was significantly higher than these of paired adjacent non-HCC tissues (0.20±0.13, *P*<0.001). So was found in Nanog of cancerous and paired normal tissues (0.68±0.12 *versus* 0.22±0.12, *P*<0.001; Figure 6b). When cutting off with mIOD, 16 of 45 patients (35.6%) were categorized into Sp1 low-expressed group and the remaining (29/45, 64.4%) into Sp1 high-expressed group. Similarly, 44.4% (20/45) of patients were sorted into Nanog low-expressed group and 55.6% (25/45) were Nanog high-expressed group. As shown in Table 1, Sp1 is positively associated with tumor diameters (*P*=0.033), whereas Nanog is related with BCLC stage (*P*=0.040) and microvascular invasion (*P*=0.045). Lower overall survivals were found in both Sp1 (Log-rank, *P*=0.001) and Nanog (Log-rank, *P*=0.005) high-expressed patients than those in low-expressed ones, when analyzed with Kaplan–Meier survival assays (Figure 6c). Thus, Sp1 or/and Nanog were unfavorable correlation coefficient on HCC prognoses, suggesting that they were possibly used as poor prognostic biomarkers in HCC patients.

## Discussion

CSCs, although a small subset in cancer population, have been thought as the initiating cells not only in tumorigenesis, metastasis and recurrence^24^ but also in tumor heterogeneity and drug resistance.^7^ To date, CSCs have been identified in most solid tumors, including colon,^25^ breast^26^ and liver cancer.^27^ Like ESCs, CSCs were often recognized by specific phenotypes. In HCC, many CSCs biomarkers had been verified, among which EpCAM and PROM1 (CD133) were the two familiar ones.^17, 28, 29^ High EpCAM and CD133 expressed liver cancer cells usually possessed tumor-initiating or stemness-like property,^17^ and reprogrammed by a panel of pluripotency transcript factors, such as Sox2, Nanog, Klf4, Oct4 and so on.^30, 31^ EpCAM itself was regulated directly by Oct4^17^ and CD133 regulated by numerous factors as Nanog, Oct4, Sox2 and c-Myc.^21, 32, 33, 34^ These molecules consist of imprinted signatures of HCC CSCs.

Recently, miRNAs have been proven as stemness regulators in many cancers. Different from the classical extracellular stimulators as Notch, Hedgehog and so on,^35^ miRNAs usually regulate the stemness of CSCs endogenously. Previously, we verified that miR-612 was a main adjuster of EMT-associated stem cell-like traits of HCC.^16^ As multiple targets could be modulated simultaneously by one given miRNA, theoretically, it is much more likely that new uncovered ways of miR-612 existed in stem-like property regulation in HCC.

By Flow cytometer analysis, we easily found that EpCAM and CD133 were negatively regulated by miR-612. And a significant inverse correlation between miR-612 and two biomarkers was confirmed once again in this study. The results, together with our previous findings, convince us that miR-612 was an important suppressor of HCC CSCs. To uncover underlying mechanism, Nanog, Klf4, Sox2 and Oct4, four main ESCs transcript factors,^36, 37^ were selected and monitored in miR-612-i HepG2 and miR-612-o HCCLM3 cells. In the context, Nanog was the only one inhibited by miR-612 markedly. Owing to no typical seed sequence in the 3'-UTR of Nanog, an indirect role of miR-612 was naturally surveyed next. Sp1, a transcription factor of KLF family, arouse us much interested in that it was able to promote murine Nanog transcription^21^ and it had a predicted miR-612 binding site by several programs. Therefore, we explored the possibility of Sp1-mediated Nanog expression in HCC. As expected, Sp1 indeed specifically interacted with *nanog* promoter and facilitated its transcription. And Sp1 was a direct downstream target of miR-612 as confirmed by luciferase assays. More interestingly, they both overexpressed in HCC tissue and had a low-correlation relationship. The results implied that Sp1/Nanog signaling pathway could be modulated by miR-612. To further evaluate their clinical signatures, Sp1 and Nanog were artificially divided into high- and low-expressed groups with mIOD, although both were faintly expressed only in a few patients. In the context, Sp1 is positively associated with tumor diameters, whereas Nanog is significantly related with BCLC stage and microvascular invasion. And high levels of both proteins in tumor tissues had poor prognostic values on overall survival of HCC patients. All these were in keep with other previous observations.^38^ However, the differences between Sp1 and Nanog in the process of HCC progression were noticed, in that the former probably had a more wide function as tumor size regulation on most side population, while the latter had a relative narrow role on tumor invasion in small subset of HCC population.

In conclusion, miR-612 does a key regulator on HCC stemness by Sp1/Nanog axis. It probably is a new biomarker in HCC progression, although a multicenter-clinical trial needed in the future.

## Material and Methods

### Cell lines and cell culture

Human HCC cell line, HCCLM3, was established at the Liver Cancer Institute, Zhongshan Hospital, Fudan University, Shanghai, China.^39, 40^ HepG2 cell line was purchased from the Shanghai Cell Bank, Chinese Academy of Sciences (CAS). HCCLM3 cells have a relatively high metastatic potential and express a low endogenous level of miR-612, whereas HepG2 cells have a lower metastatic potential and express a higher endogenous level of miR-612.^15, 16^ All these cells were cultured under standard conditions, DMEM (GE, Logan, UT, USA) supplemented with 10% FBS (GE), and routinely maintained in a humidified incubator at 5% CO_2_ at 37 °C.^15^

### Oligonucleotides and transfection

The oligonucleotides including miR-612 hairpin inhibitor, mimic and NC (Thermo Fisher Scientific, Cleveland, OH, USA) for inhibition and restoration of miR-612 were used in this study. And four GV248 plasmid particles containing short hairpin sequences targeting the human *sp1* gene as well as the NC plasmids were purchased from GeneChem (GeneChem, Shanghai, China). All these constructs and oligonucleotides were transfected into HCC cells using Lipofectamine 2000 according to the product manual (Thermo Fisher Scientific, Waltham, MA, USA).^15^ Four shRNA sequences were shown in Supplementary Table S1.

### RNA extraction and real-time PCR assays for mRNA detection

Total RNA was extracted from cultured cells with Trizol Reagent (Thermo Fisher Scientific). The quality and integrity of RNA were evaluated via A260/A280 ratio, and then 1 *μ*g of total RNA was used for first-strand DNA synthesis. Real-time PCR was performed in triplicate by the SYBR Green PCR method using an All-in-One miRNA qPCR Detection kit (GeneCopoeia, Rockville, MD, USA). The forward primers of has-miR-612 and U6 small nuclear RNA (U6) were synthesized as Supplementary Table S2. The common reverse primer was purchased from the same company. For mRNA detection, 1 *μ*g of total RNA was used for complementary DNA synthesis with a PrimeScript RT reagent kit (Takara Bio, Kyoto, Japan). Real-time PCR was performed in triplicate using SYBR Premix Ex Taq (Takara Bio). The primers for the genes of interest (EpCAM, CD133, Nanog, Klf4, Sox2, Oct4 and Sp1) were synthesized by Sangon Biotech (Sangon Biotech, Ltd, Shanghai, China) as Supplementary Table S2. The U6 and GAPDH were used as internal control for miRNAs and mRNAs assays, respectively. The threshold cycle (Ct) values were analyzed using the comparative Ct (−ΔCt) method.^41^ The level of targets was obtained by normalizing to the endogenous reference and relative to a control.

### Protein levels detected by western blot analysis

Lysates were obtained from cultured cells with a mixture of ProteoJET Mammalian Cell Lysis Reagent (Thermo Fisher Scientific) and PMSF (Roche, Basel, Switzerland). About 20 *μ*g protein was extracted from each sample, separated by 10%SDS-PAGE and transferred onto polyvinylidene fluoride membranes. After being blocked in 5% bovine serum albumin, the interested protein was probed with antibodies against human EpCAM (1 : 1000; Abcam, Cambridge, MA, USA), CD133(1 : 1000; ProteinTech Group, Chicago, IL, USA), Nanog (1 : 1000; Cell Signaling Technology, Danvers, MA, USA), Klf4 (1 : 1000; Cell Signaling Technology), Sox2 (1 : 1000; ProteinTech Group), Oct4 (1 : 1000; ProteinTech Group), Sp1 (1 : 200;Cell Signaling Technology), GAPDH (1 : 3000, Abcam), and incubated with goat anti-rabbit or anti-mouse IgG (1 : 10 000 for both; Jackson ImmunoResearch Laboratories, West Grove, PA, USA), and detected with enhanced chemiluminescence reagents (Thermo Fisher Scientific). The bands were visualized using 1-stepTM NBT/BCIP reagents (Thermo Fisher Scientific) and detected by Tanon 5200 automatic chemiluminescence image analysis system (Tanon, Shanghai, China).

### Luciferase reporter assay

The binding sites for miR-612 in the 3′ UTR sequence of Sp1 were cloned into the pMIR, and the original sequence of *nanog* promoter with Sp1 binding sites were cloned into the pGL4.10 GLO Dual-Luciferase Expression Vector (Promega, Madison, WI, USA). These constructs with mutant and blank plasmids were co-transfected into target cells in 96-well plates together with miR-612 mimic or Sp1 overexpression plasmid using Lipofectamine 2000. Luciferase activity was measured 24 h after transfection using the Dual-Luciferase Reporter Assay System (Promega). The levels of firefly luciferase activities were obtained by normalizing to Renilla luciferase activities and relative to a control, as previously reported.^42^

### Chromatin immunoprecipitation

HCCLM3 and HepG2 cells were cultured, fixed (1% formaldehyde), washed, harvested and lysed, followed by sonicating to produce chromatin of primarily mono-nucleosomal size. Immunoprecipitation was performed overnight with anti-Sp1 or anti-IgG antibodies. Protein-DNA complexes were recovered using protein G agarose beads, washed, and then eluted. Cross-links were reversed at 65 °C overnight, and DNA was purified using reagents provided in the EZ-ChIP Chromatin Immunoprecipitation Kit (Millipore, Bedford, MA, USA). The immunoprecipitated DNA was amplified by PCR for sequences containing Sp1-binding sites. Ten paired primers were designed against 3000 nt further upsteam of *nanog* ORF (Supplementary Table S3).

### Patient selection and TMA construction

Forty-five patients with primary HCC who underwent curative liver resection in Zhongshan Hospital (Shanghai, China) between July 2011 and April 2013 were included. The follow-up information was updated until 1 January 2016. The mean follow-up interval was 33.4 months (ranging from 12 to 54 months). Until the last follow-up, 12 patients (26.7%) were alive, 33 (73.3%) were dead. Forty-five HCC tissues and adjacent normal liver tissues were obtained and made into TMA according to the previously published method.^43^ All procedures were approved by the Zhongshan Hospital Research Ethics Committee. Informed consent was obtained from each patient according to regulations set forth by the Ethics Committee.

### Immunohistochemical staining

Immunohistochemistry for the target molecules was performed on tissue microarray. The slides were probed with a primary antibody against Sp1 (1 : 2000; Cell Signaling Technology), Nanog (1 : 1000; Cell Signaling Technology) and then incubated with horseradish peroxidase-conjugated IgG (1 : 500; Thermo Fisher Scientific), and the proteins *in situ* were visualized with 3, 3′-diaminobenzidine. The intensity of positive staining was measured with integrated optical density (IOD) as previously described.^44^

### Immunofluorescence staining

Cells were collected from the monolayer culture using trypsin, scattered onto glass bottom dish, and then fixed with 4% paraformaldehyde. Next, the cells were incubated with the EpCAM antibody (1 : 250) and CD133 antibody (1 : 100) in 4 °C overnight followed by Alexa Fluor 488-donkey anti-rabbit IgG (1 : 250; ProteinTech Group) and Alexa Fluor 594-conjugated goat anti-mouse IgG (1 : 250; ProteinTech Group) for 1 h in the dark. The cell nuclei were counterstained with DAPI. Images were obtained under the Laser Scanning Confocal Microscope.

### Tumorsphere assay

Five hundred cells were plated into ultra-low attached 96-well plates, cultured in 200 *μ*l DMEM/F12 media (Sigma-Aldrich, St. Louis, MO, USA) with B27 supplement (Thermo Fisher Scientific), antibiotics, 20 ng/ml of epidermal growth factor (PeproTech, Rocky Hill, NJ, USA), 20 ng/ml of basic fibroblast growth factor (PeproTech). After 4–5 days, equal fresh media was added. Cells were incubated for 2 weeks, and spheres with diameter >50 *μ*m were counted.

### Statistical analysis

Data were analyzed using GraphPad Prism 6.0 software or Statistical Program for Social Sciences software 19.0 (SPSS, IBM, Chicago, IL,USA). Quantitative variables were expressed as means±S.D. and analyzed by one-way ANOVA, Student's *t*-test, Kruskal–Wallis test, or Mann-Whitney *U*-test. Qualitative variables were compared using Pearson *χ*^2^-test or Fisher exact test. Overall survival was estimated using Kaplan–Meier method, and the difference in survival was evaluated by log-rank tests. Results were considered statistically significant at *P<0.05, **P<0.01, ***P<0.001.

## Figures and Tables

**Table 1:** Associations between Sp1, Nanog expression and clinicopathological characteristics of HCC patients

**Variable**	**Cases (*****n*****=45)**	**Relative Sp1 expression**			**Relative Nanog expression**		
		**Low (*****n*****=16)**	**High (*****n*****=29)**	***P***-**value**^a^	**Low (*****n*****=20)**	**High (*****n*****=25)**	***P-*****value**^a^
*Age(year)*				*0.577*			*0.502*
≤54	20	8	12		10	10	
>54	25	8	17		10	15	
							
*Sex*				*0.430*			*0.786*
Male	34	11	23		16	18	
Female	11	5	6		4	7	
							
*HBsAg*				*0.766*			*0.938*
Negative	11	3	8		5	6	
Positive	34	13	21		15	19	
							
*HBeAg*				*0.474*			*1.000*
Negative	40	13	27		18	22	
Positive	5	3	2		2	3	
							
*AFP (ng/ml)*				*0.539*			*0.783*
≤20	17	7	10		8	9	
>20	28	9	19		12	16	
							
*Tumor diameter (cm)*				***0.033***			*0.288*
<3	12	8	4		7	5	
3–5	14	3	11		4	10	
>5	19	5	14		9	10	
							
*Tumor number*				*0.988*			*0.885*
Single	31	11	20		14	10	
Multiple	14	5	9		6	15	
							
*BCLC*				*0.227*			***0.040***
A	9	5	4		7	2	
B	15	6	9		7	8	
C	21	5	16		6	15	
							
*Microvascular invasion*				*0.124*			***0.045***
No	24	11	13		14	10	
Yes	21	5	16		6	15	
							
*Ascites*				*0.531*			*0.192*
No	43	16	27		18	25	
Yes	2	0	2		2	0	
							
*Differentiation*				*0.207*			*0.458*
I	1	1	0		0	1	
II	31	12	19		15	16	
III	13	3	10		5	8	
							
*Cirrhotic nodule (cm)*				*0.318*			*0.091*
0	7	3	4		4	3	
0.1–0.3	15	5	10		3	12	
0.4–0.6	19	5	14		10	9	
>0.6	4	3	1		3	1	

a Qualitative variables were compared using *χ*^2^-test. Bold data indicates statistical significance (*P*<0.05)

## Abbreviations

HCC: hepatocellular carcinoma cells
CSC: cancer stem cell
EpCAM: epithelial cell adhesion molecule
miRNA: microRNA
NOD/SCID: non-obese diabetic/severe combined immunodeficient
WT: wild type
NC: negative control
ESC: embryonic stem cells
Oct4: octamer-binding transcription factor 4
Sox2: SRY (sex determining region Y)-box 2
Klf4: Kruppel-like factor 4
mRNA: messenger RNA
Chip-PCR: chromatin immunoprecipitation-polymerase chain reaction
ORF: open-reading frame
nt: nucleotide
DNA: deoxyribonucleic acid
shRNA: short hairpin RNA
PCR: polymerase chain reaction
mIOD: median value of integrated optical density
UTR: untranslated region
FBS: fetal bovine serum
