# Supplementary material for: miR-612 suppresses stem cell-like property of hepatocellular carcinoma cells by modulating Sp1/Nanog signaling
Source: Cell Death Dis. 2016 Sep 29;7(9):e2377–. doi: 10.1038/cddis.2016.282 (PMC5059880; doi:10.1038/cddis.2016.282)
Supplement: Supplementary Information [file cddis2016282x1.doc]

Table S1. The sequences of four shRNAs targeting *sp1*

| shRNA1 | 5’-GCTGGTGGTGATGGAATACATCTCGAGATGTATTCCATCACCACCAGC-3’ |
| --- | --- |
| shRNA2 | 5’-CCCAAGTTTATTTCTCTCTTACTCGAGTAAGAGAGAAATAAACTTGGG-3’ |
| shRNA3 | 5’-CCACTCCTTCAGCCCTTATTACTCGAGTAATAAGGGCTGAAGGAGTGG-3’ |
| shRNA4 | 5’- CCAGGTGCAAACCAACAGATTCTCGAGAATCTGTTGGTTTGCACCTGG-3’ |

Table S2. Sequence of PCR primers used in this study

| has-miR-612 | | 5’- GCAGGGCTTCTGAGCTCCTTAA -3’ |
| --- | --- | --- |
| U6 small nuclear RNA (U6) | | 5’- CAAATTCGTGAAGCGTTCCATAT -3 |
| EpCAM | forward | 5’-CTCGCGTTCGGGCTTCT-3’ |
|  | reverse | 5’-TGTAGTTTTCACAGACACATTCTTCCT-3’ |
| CD133 | forward | 5’-GTGTCCTGGGGCTGCTGTTTA-3’ |
|  | reverse | 5’-CCATTTTCCTTCTGTCGCTGG -3’ |
| Nanog | forward | 5’- CGATCTCCTGACCTTGT-3’ |
|  | reverse | 5’-CACGCCTGTAAATCCCA-3’ |
| Klf4 | forward | 5’-GATGATGCTCACCCCACCTT-3’ |
|  | reverse | 5’-TGTGCCTTGAGATGGGAACTC-3’ |
| Sox2 | forward | 5’-ATCAGGAGTTGTCAAGGCAGAG-3’ |
|  | reverse | 5’-AGAGGCAAACTGGAATCAGGA -3’ |
| Oct4 | forward | 5’-AAGCGATCAAGCAGCGAC-3’ |
|  | reverse | 5’-GGAAAGGGACCGAGGAGTA-3’ |
| Sp1 | forward | 5’-GTGGGAAGCCAAGACAACAT-3’ |
|  | reverse | 5’-GGGAGGAGGAAGACCATTCT-3’ |
| GAPDH | forward | 5’-CACCATGAAGATCAAGATCATTGC-3’ |
|  | reverse | 5’-GGCCGGACTCATCGTACTCCTGC-3’ |

Table S3. Ten paired primers designed according to the promoter of *nanog* gene

| 1-F | CAACAAGAGGGAAACTCC | 1-R | CACCAAAATGAAGATGGG |
| --- | --- | --- | --- |
| 2-F | GGATTTGGTCAGCTCCTT | 2-R | ATGATGGGTTCCAATACT |
| 3-F | TTTCTCTCATGCCTTTACCC | 3-R | CACTGCACTCCAGCCGAGG |
| 4-F | GCGTGATCTCGGCTCGCTGC | 4-R | GACTTTAGATTTTTTGGAAC |
| 5-F | AGATAGCTTCCTAAACCT | 5-R | CTTAATCAGCACAGTTTG |
| 6-F | AGAGACAGGAGGGCAAGT | 6-R | ATTCTCCTCTGCACTCTG |
| 7-F | GAGTCAAAGAGTTTTGTCT | 7-R | AGCTTCTTCCCAGGTCTG |
| 8-F | AAAGAGCCAGAGGGAAAA | 8-R | GCACCTTAAATTCCTGAG |
| 9-F | ATGCATCCCCCACCCCCC | 9-R | TGGCCCACCCCTGTAATCCC |
| 10-F | CCGCGCCCGGCCTTTTTCTT | 10-R | GGCTCTATCACCTTAGAC |
