# Supplementary material for: miR-612 suppresses stem cell-like property of hepatocellular carcinoma cells by modulating Sp1/Nanog signaling
Source: Cell Death Dis. 2016 Sep 29;7(9):e2377–. doi: 10.1038/cddis.2016.282 (PMC5059880; doi:10.1038/cddis.2016.282)
Supplement: Supplementary Information [file cddis2016282x2.doc]

Detailed Attribution of Authorship

Yang Liu, Dong-Li Liu, Li-Li Dong and Wei-Zhong Wu conceived and designed the study. Yang Liu, Dong-Li Liu, Li-Li Dong performed the experiments including IHC, RT-PCR, Western blotting and Tumorsphere assay. Yang Liu, Dong-Li Liu performed luciferase reporter assays. Li-Li Dong performed [immunofluorescence](javascript:void(0);) staining and chromatin immunoprecipitation. Duo Wen, Dong-Min Shi selected patients and construction TMA. Yang Liu, Dong-Li Liu, Li-Li Dong and Wei-Zhong Wu analyzed the data and prepared the manuscript. Zhou Jian, Jia Fan participated in the study design. All authors read and approved the final manuscript.
